# COVID-19 Pneumonia: An Emerging Cause of Syndrome of Inappropriate Antidiuretic Hormone

**DOI:** 10.7759/cureus.8841

**Published:** 2020-06-26

**Authors:** Muhammad Mubbashir Sheikh, Ejaz Ahmad, Hafiz Muhammad Jeelani, Adeel Riaz, Ahmad Muneeb

**Affiliations:** 1 Oncology, Northwestern University Feinberg School of Medicine, Chicago, USA; 2 Internal Medicine, Mayo Hospital, Lahore, PAK; 3 Internal Medicine, Nishtar Hospital, Multan, PAK; 4 Internal Medicine, Rosalind Franklin University of Medicine and Science, McHenry, USA; 5 Anesthesiology and Critical Care, District Headquarter Hospital, Sahiwal, PAK; 6 Internal Medicine, Allied Hospital / Faisalabad Medical University, Faisalabad, PAK

**Keywords:** siadh, hyponatremia, covid-19

## Abstract

Syndrome of inappropriate antidiuretic hormone (SIADH) is the leading cause of hyponatremia. We, herein, report a case of a patient with coronavirus disease-2019 (COVID-19) who developed sudden exertional dyspnea and hypoxia and was found to be hyponatremic. A diagnosis of SIADH was made due to COVID-19 pneumonia. The patient was managed conservatively with a significant improvement during the course of hospitalization and on follow-up.

## Introduction

Since December 2019, the outbreak of coronavirus disease 2019 (COVID-19) from Wuhan, China, has not only caused global social and economic determinants but also profoundly impacted the current context of scientific research. The disease has been reported to involve many organs, particularly, lungs, kidneys, and is linked to a myriad of complications resulting in high mortality across the globe [1]. As of May 28, 2020, more than 6,000,000 confirmed cases and more than 350,000 deaths have been documented worldwide [2]. The symptoms of COVID-19 are mostly dependent on the age and the immunity of the patients [3]. Most common symptoms encountered during clinical practice include fever (88.7%), cough (57.7%), dyspnea (45.6%), and diarrhea (3.8%) [4]. Recently, hyponatremia has been reported as a common electrolyte disorder in COVID-19 patients [5-6]. Similar to our patient, the limited data have shown COVID-19 pneumonia as an underlying cause of syndrome of inappropriate antidiuretic hormone secretion (SIADH) induced hyponatremia [7-8]. SIADH is a diagnosis of exclusion characterized by dilutional hyponatremia in the absence of any identifiable renal pathology, diuretics intake, or any other no-osmotic stimulating factors for antidiuretic hormone (ADH) production [9-10]. Our case report adds to the ongoing research that proposes the significance of recognizing the fundamental etiology for hyponatremia in patients with COVID-19. As treatment can vary for hyponatremia in different underlying conditions, it is of paramount importance to have the proper management to prevent consequential complications.

## Case presentation

A 37-year-old male with no significant past medical history presented to the ED with complaints of fever, dry cough, malaise for three days, and progressively worsening dyspnea for one day. No additional symptoms were reported by the patient. Social history was negative for smoking, alcohol, or illicit drugs. Clinically, the patient was alert and oriented to time, place, and person. Physical examination revealed a temperature of 101°F, normotensive, respiratory rate (RR) of 28 breaths per minute, and heart rate (HR) of 115 beats per minute. His oxygen saturations were 88% on ambient air. Further, a focused respiratory exam revealed reduced air entry and bilateral scattered wheezing on auscultation. The rest of the pertinent physical examination was negative for pedal edema, hepatomegaly, jugular venous distension (JVD), or any heart murmur.

**Figure 1:**
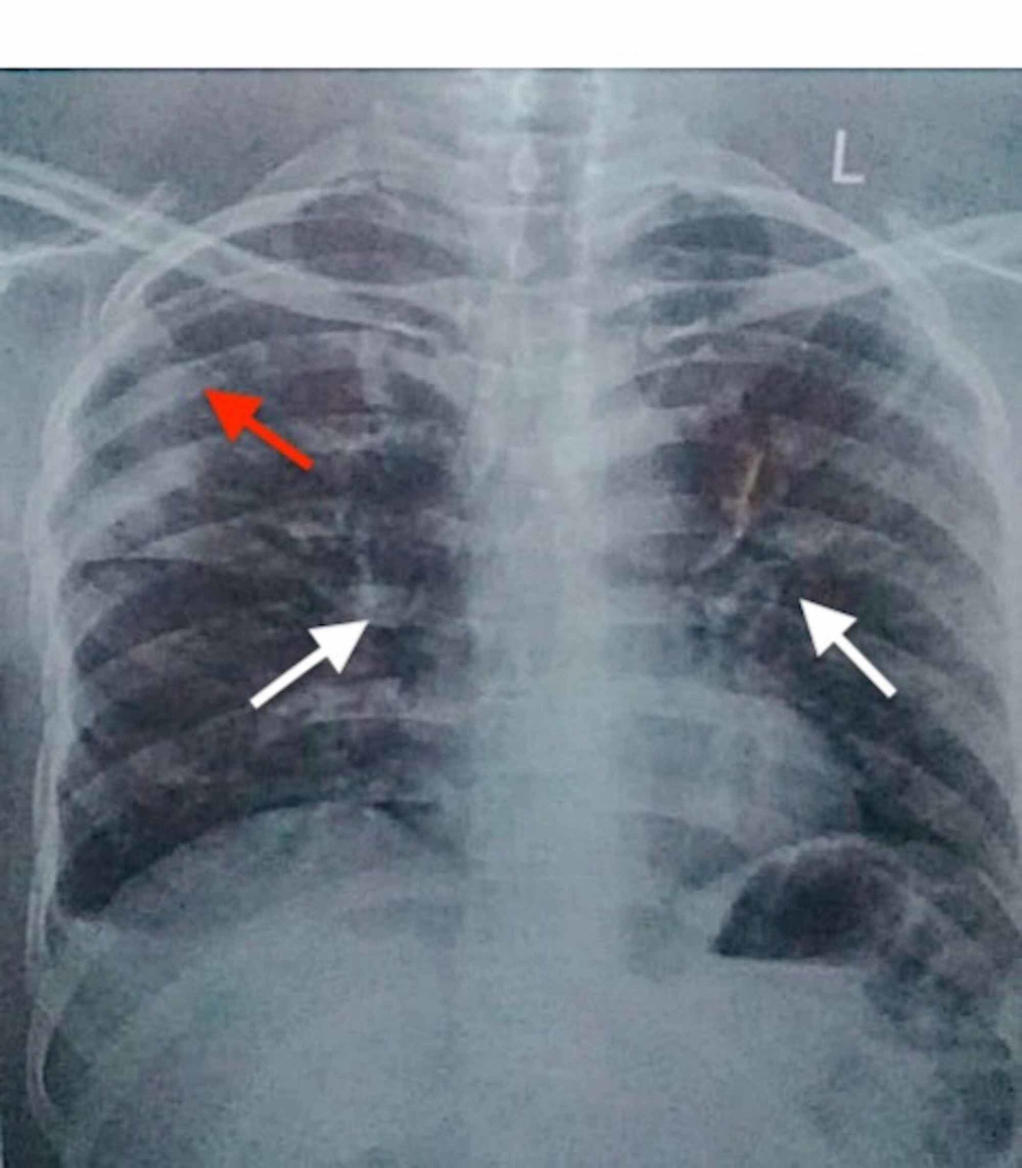
Chest radiograph showing pneumonitis patch in the peripheral region of right mid zone (red arrow) and bilateral alveolar infiltrates (white arrows).

Laboratory investigations at the time of admission revealed significant low serum sodium (Na) level of 118 meq/L (normal range: 135-145 meq/L) with a serum osmolality of 239 mOsm/kg (normal range: 285-294 mOsm/kg), a white blood cell (WBC) count of 7.92 cells/mm3 (normal range: 4-11 cells/mm3) with 86% neutrophils (normal range: 40%-75%) and 10% lymphocytes (normal range: 18%-42%). Serum potassium, creatinine, calcium and magnesium levels, liver function tests, and coagulation profile including prothrombin time (PT), activated partial thromboplastin time (aPTT) were within the normal range. Chest X-ray (CXR) showed a pneumonitis patch in the peripheral region of the right mid zone and bilateral alveolar infiltrates, highly suggestive of COVID-19 pneumonia (Figure 1). Nasopharyngeal swab via real-time reverse transcriptase-polymerase chain reaction analysis (RT-PCR) confirmed COVID-19. Further, the workup for euvolemic hyponatremia revealed urine osmolality of 338 mOsm/kg (normal range: 50-1200 mOsm/kg) and urine spot sodium 45 mmol/L (normal range: 54-190 mmol/L). Additionally, the normal range levels of thyroid-stimulating hormone and cortisol exclude adrenal pathology as a cause of hyponatremia. A diagnosis of COVID-19 pneumonia and SIADH was made.

Initially, the patient was managed with appropriate oxygenation, intravenous moxifloxacin, and acetaminophen on the lines of community-acquired pneumonia.

Fluid restriction of 1 L/day (1000 mL/day) was done. After 12 hours of confirmed suspicion of novel coronavirus i.e., severe acute respiratory syndrome coronavirus (SARS-Cov-2), the patient was transitioned to COVID-19 designated isolation ward. Serum sodium levels repeated at 24, 48, and then 72 hours revealed levels of 123, 129, and 136 mEq/L, respectively. Concerning COVID-19 pneumonia, moxifloxacin was stopped, azithromycin 500 mg once daily for five days, zinc sulfate 50 mg, and vitamin C 500 mg (ascorbic acid) for 14 days each were administered. During hospitalization, a marked improvement in respiratory status with decreased oxygen requirement was observed. At two weeks of follow-up, labs (WBC count of 6.18 with 65% neutrophils and 18% lymphocytes, and serum Na level of 139 mEq/L, respectively) and CXR (accentuated bronchovascular markings observed bilaterally with no acute exudative lesion, lymphadenopathy, or pleural fluid) depicted the improved baseline health status of the patient.

## Discussion

Hyponatremia is a frequently encountered electrolyte disorder in hospitalized patients and a predictor of overall mortality and morbidity [11]. SIADH has been identified as a predominant cause of hyponatremia in up to 50% of cases [12]. Table 1 lists the diagnostic criteria for SIADH [9-10]. 

**Table 1:** Diagnostic criteria of SIADH. SIADH: syndrome of inappropriate antidiuretic hormone; ADH: antidiuretic hormone *Essential diagnostic features; **Supplemental diagnostic features

Essential and supplemental diagnostic features
Serum osmolality < 280 mOsm/kg*
Increased urinary sodium (>40 mmol/L with normal dietary salt intake)*
Euvolemic*
Normal thyroid and adrenal function*
No recent use of diuretics*
Fractional sodium excretion >1%; fractional urea excretion >55%**
Elevated plasma ADH levels, despite the presence of hypotonicity and clinical euvolemia**

Common lung pathologies such as pulmonary malignancy, severe obstructive lung disease, acute respiratory failure, and pneumonia are the prevalent disorders that can lead to SIADH [9]. Among viral infections, influenza has been identified as an underlying cause of SIADH [13-14]. In recent times, COVID-19 pneumonia has emerged as a causative factor for SIADH. The pathogenesis for the development of SIADH in COVID-19 pneumonia per preliminary reports is due to the production of certain proinflammatory cytokines, particularly, IL-6. These cytokines increase the ADH production via twofold mechanisms: firstly, by direct stimulation of nonosmotic release of ADH, and secondly by the direct insult of alveolar basement membrane resulting in activation of hypoxic pulmonary vasoconstriction pathway that can lead to increase ADH production [15-18]. Thus, suggesting endogenous nonosmotic ADH production. However, further studies are needed to extrapolate this mechanism.

The effective management of SIADH depends on the identification of underlying etiologies, determination of serum sodium (Na) levels, and urine osmolality. Table 2 lists the classification of hyponatremia with common underlying conditions [12]. Close monitoring of Na levels and urine osmolality will dictate the rate of fluid resuscitation and will also help to avoid exacerbation of respiratory distress especially in patients with pulmonary infections or disorders [19]. In hyponatremia either acute (<48 hours) or chronic (>48 hours) fluid correction should be done cautiously to avoid the precipitous drop of Na levels of >8 mEq/L over a period of 24 hours in order to prevent the risk of associated complications [20]. General treatment includes the administration of aggressive hypertonic saline (acute hyponatremia) and fluid restriction to less than water excretion in (chronic hyponatremia) or asymptomatic patients with SIADH [12]. Other options that can be utilized in SIADH are loop diuretics, vasopressin receptor antagonists, demeclocycline, and oral salt tablets [19-20]. Fluid restriction along with supportive management of COVID-19 was the mainstay to correct SIADH in our patient.

**Table 2:** Hyponatremia classification with common underlying etiologies. ADH: antidiuretic hormone; SIADH: syndrome of inappropriate antidiuretic hormone; UNa: urinary sodium *Urine sodium excretion - low; **Urine sodium excretion - high

Hypervolemia (UNa excretion - low, plasma renin - high)	Hypovolemia (UNa excretion - low or high, plasma renin - high)	Euvolemia (UNa excretion - low or high, plasma renin - low)
Congestive heart failure*	Bleeding*	SIADH**
Cirrhosis*	Diuretic use*	Primary polydipsia*
Nephrotic syndrome*	Diarrhea*	Hypothyroidism*
	Adrenal insufficiency**	

## Conclusions

Our case emphasizes the consideration of SIADH in hospitalized COVID-19 patients having hyponatremia. The correct identification of the underlying etiology in hyponatremia through detailed workup is critical for curtailing the length of hospital stay, inappropriate treatment, and debilitating morbidity in the current pandemic. In summary, COVID-19 pneumonia is an evolving cause of SIADH; however, future studies are warranted to explore this association.
